# Detection and Differentiation of SARS-CoV-2, Influenza, and Respiratory Syncytial Viruses by CRISPR

**DOI:** 10.3390/diagnostics11050823

**Published:** 2021-05-01

**Authors:** Huifen Zhou, Jen-Hui Tsou, Molangur Chinthalapally, Hongjie Liu, Feng Jiang

**Affiliations:** 1 Department of Pathology, University of Maryland School of Medicine, 10 S. Pine St., Baltimore, MD 21201, USA; Huifen.Zhou@som.umaryland.edu (H.Z.); JTsou@som.umaryland.edu (J.-H.T.); 2 Environmental Science and Technology, College of Agriculture and Natural Resources, University of Maryland, College Park, MD 20742, USA; chinthalapallyhdrao@yahoo.com; 3 Department of Epidemiology and Biostatistics, School of Public Health, University of Maryland, College Park, MD 20742, USA; hliu1210@umd.edu

**Keywords:** detection, SARS-CoV-2, influenza, respiratory syncytial viruses

## Abstract

SARS-CoV-2, influenza, and respiratory syncytial viruses (RSVs) cause acute respiratory infections with similar symptoms. Since the treatments and outcomes of these infections are different, the early detection and accurate differentiation of the viruses are clinically important for the prevention and treatment of the diseases. We previously demonstrated that clustered regularly interspaced short palindromic repeats (CRISPR) could rapidly and precisely detect SARS-CoV-2. The objective of this study was to develop CRISPR as a test for simultaneously detecting and accurately distinguishing the viruses. The CRISPR assay with an RNA guide against each virus was performed in the reference standards of SARS-CoV-2, influenza A and B, and RSV. The CRISPR assay had a limit of detection of 1–100 copies/µL for specifically detecting SARS-CoV-2, influenza A and B, and RSV without cross-reaction with other respiratory viruses. The validation of the test in nasopharyngeal specimens showed that it had a 90–100% sensitivity and 100% specificity for the detection of SARS-CoV-2, influenza A and B, and RSV. The CRISPR assay could potentially be used for sensitive detection and specific differentiation of the respiratory viruses.

## 1. Introduction

Severe acute respiratory syndrome coronavirus 2 (SARS-CoV-2) causes coronavirus disease 2019 (COVID-19), the subject of an ongoing global pandemic [1]. The concurrent infections of SARS-CoV-2 with other pathogens, such as influenza viruses and other seasonal coronaviruses, could make the epidemic even worse. SARS-CoV-2, influenza, and respiratory syncytial viruses (RSV) can cause acute respiratory infections with similar symptoms. However, the treatments and outcomes of infections by the viruses are different. Therefore, rapid detection and accurate differentiation of the viruses are clinically important for efficient prevention and treatment of the diseases.

Reverse transcription polymerase chain reaction (RT-PCR)-based assays have been developed for the detection of SARS-CoV-2, influenza viruses, and other respiratory viruses [2]. For instance, the Xpert four-in-one assay can simultaneously detect SARS-CoV-2, influenza, and RSV in upper respiratory tract specimens [3]. However, the PCR-based assay is cost and labor intensive and requires expensive machinery [4]. Clustered regularly interspaced short palindromic repeats (CRISPR) is a family of DNA sequences found within the genomes of prokaryotic organisms [5]. A CRISPR-associated (Cas) immune system has been applied in molecular biology to target and cleave specific nucleic acid sequences, which is commonly used in gene editing [6]. Furthermore, Cas proteins are shown to be activated and unleash nonspecific endoribonuclease activity, causing the cleavage of DNA or RNA and thus providing a novel diagnostic approach for the detection of nucleic acid [7,8,9,10,11]. For instance, using Cas12a, Chen et al. developed a method termed the DNA endonuclease-targeted CRISPR trans reporter (DETECTR) for nucleic acid detection [9]. Our previous study has demonstrated that CRISPR-Cas12a can detect nucleic acids of exogenous viruses, such as human papillomavirus, in the raw plasma of cervical cancer patients without requiring RNA extraction [12]. Our research has also demonstrated that the CRISPR-based test can rapidly and sensitively detect endogenous DNA mutations of EGFR and KRAS in plasma and tissue specimens of lung cancer patients [13,14]. Recently, we developed CRISPR-Cas12a as a rapid test for sensitive detection of SARS-CoV-2 [15]. In this study, we investigated if the CRISPR-Cas12a test could simultaneously detect and precisely distinguish different respiratory viruses.

## 2. Materials and Methods

### 2.1. Reference RNA Standards of Viruses

We obtained quantitative genomic RNA of the fully heat-inactivated influenza A virus (H1N1) strain A/PR/8/34 (ATCC VR-95DQ) (ATCC, Manassas, VA, USA), influenza B virus (Victoria) strain B/Florida/78/2015 (ATCC VR-1931) (ATCC), RSV strain 9320 (ATCC VR-955D) (ATCC), SARS-CoV-1 strain 229E (ATCC VR-740DQ) (ATCC), Bocavirus (HBoV) strain C (ATCC VR-3251SD) (ATCC), and Echovirus 4 strain Pesascek (ATCCVR-1734D) from ATCC (ATCC). We also obtained an NR-52349 sample from BEI Resources (ATCC), which contained inactivated SARS-CoV2 diluted into lung carcinoma cells (A549; ATCC^®^ CCL-185™). Genomic RNA of each virus was serially diluted to obtain a tenfold gradient dilution from 10^4^ to 0.1 copies/µL to act as a standard for testing the analytical performances of the tests.

### 2.2. Oligonucleotides and RT-Recombinase Polymerase Amplification Reaction (RT-RPA)

Oligonucleotides of primers were synthesized by Integrated DNA Technologies (IDT, Coralville, IA) (Table 1). Forward and reverse primers were designed and used for amplification of the RNase P gene, which acted as an internal positive control (Table 1). The RT-RPA reaction was run for isothermal amplification of RNA targets by using TwistAmp^®^ Basic RT (TwistDx, Maidenhead, UK) according to the manufacture’s instruction. Briefly, a 50 µL reaction contained 0.48 µM forward and reverse primers (2.4 µL for each), a 29.5 µL primer-free rehydration buffer, 5 µL RNA, 14 mM magnesium acetate (MgOAc) and 1 µL supplemented with 200U/µL reverse transcriptase (ThermoFisher Scientific™, Waltham, MA, USA). The RT-PRA mixture was incubated at 42 °C.

### 2.3. Detection of the Viruses by Using CRISPR-Cas12a with a Fluorescence Plate Reader

The 3.84 µL EnGen^®^ LbaCas12a (NEB, Ipswich, MA, USA) was preassembled with gRNA (3.84 µL) in 22.32 µL of 1x NEBuffer and then mixed with 4.8 µL custom ssDNA-FQ reporter (IDT) in 25.2 µL of 1x NEBuffer [12,13,14,15]. The preassembled mixture was added directly to the 5 µL RT-RPA reactions in a volume of 20 µL and incubated at 42 °C on a Biotek fluorescence plate reader (Biotek, Winooski, VT, USA). The fluorescence kinetics was measured on the fluorescence plate reader (λex = 485 nM; λem = 535 nM) [12,13,14,15].

### 2.4. Visual Detection of the Viruses by Using CRISPR-Cas12a with a UV Light Illuminator

We used the methods described above to perform CRISPR-Cas12a reactions. As described in our previous studies [12,13,14,15], the visual detection was based on the reaction solution’s fluorescence signal, in which the tubes’ images were captured in the Bio-Rad ChemiDoc™ MP imaging system with its built-in UV channel (Bio-Rad Laboratories, Hercules, CA, USA).

### 2.5. Simultaneous Detection of Multiple Viruses by CRISPR-Cas12a in a Single Plate

**Figure 1:**
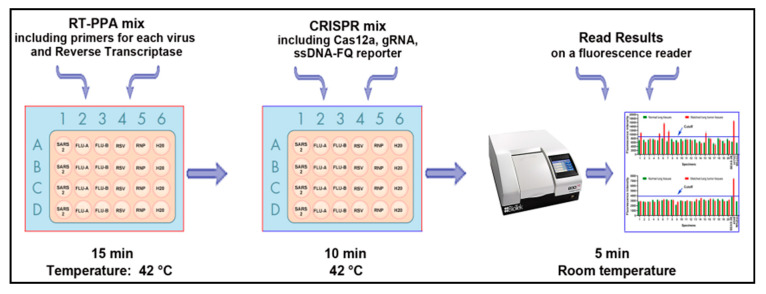
The workflow of the plate-based CRISPR for detection and differentiation of viruses.

CRISPR-Cas12a detection by a UV light illuminator only identified a single target at one time. The ability to perform multiplex detection, whereby numerous targets could be quantified simultaneously in one plate, would be practically useful. To simultaneously detect multiple viruses, we developed the workflow of a plate-based CRSIPR assay (Figure 1).

First, a 10 µL RT-RPA reaction containing 0.48 µM forward and reverse primers, a 6 µL primer-free rehydration buffer, RNA, 14 mM MgOAc, and 200U/µL reverse transcriptase was prepared.

**Figure 2:**
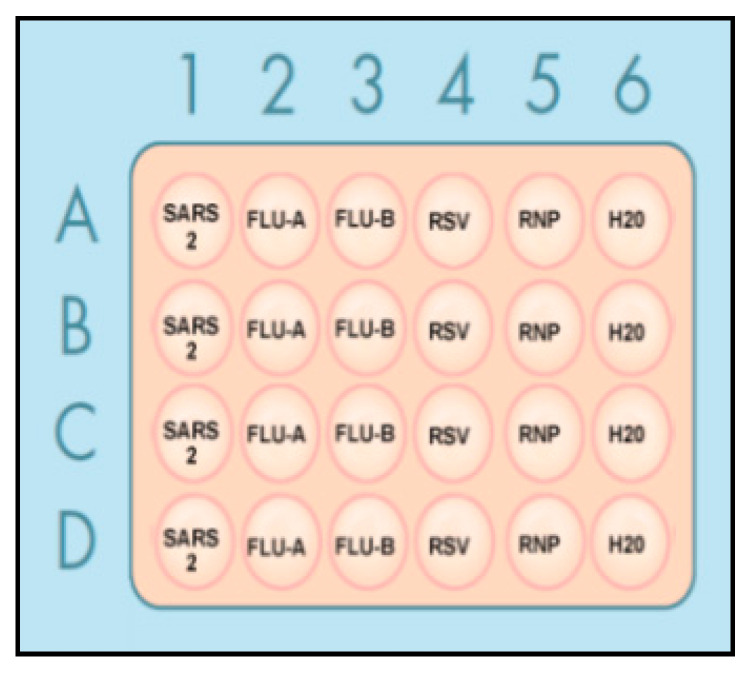
The RT-RPA mix was loaded in a 24-well plate. A 10 µL reaction containing a primer set for each target, RNA, and reverse transcriptase was added in each well in quadruplicate. Abbreviations: SARS 2 = SARS-CoV-2; FLU = influenza; and RNP = RNase P gene.

As shown in Figure 2, each RT-RPA reaction, with specific primers for the corresponding specimens, was added in a 24-well plate in quadruplicate, respectively. The RT-RPA reaction containing primers for RNase P gene was used an internal positive control, while a reaction comprising H_2_O was used as a negative control. The RT-PRA reaction was performed at 42 °C for 15 min. After RPA amplification, a 15 µL CRISPR mix containing LbaCas12a and gRNA against each virus and a custom ssDNA-FQ reporter was prepared. The CRSIPR mixes containing gRNA against the virus should be added to the individual wells, with the RT-RPA reaction amplifying the same virus. The CRISPR reaction was incubated at 42 °C for 10 min, and then the fluorescence kinetics was measured on a fluorescence plate reader for the automatic and simultaneous detection of the multiple targets.

### 2.6. RT-PCR

We used the Centers for Disease Control and Prevention (CDC) standard PCR assay to detect the viruses as described in our previous study [15]. Briefly, the RNA was amplified with a GoTaq^®^ Probe 1-Step RT-PCR system (Promega, Madison, WI, USA). The 1-step RT-PCR assay (20 µL) contained a 10 µL GoTaq Probe PCR master mix with dUTP, a 0.4 µL Go Script RT Mix for 1-step RT-PCR, 5 µL of the RNA template solution, and a 1.5 µL combined primer/probe mix, with a final concentration of 500 nM for each primer and 125 nM for the probe in the reaction. The thermal cycling protocol included 10 min at 50 °C for reverse transcription and 3 min at 95 °C for inactivation of the reverse transcriptase and initial activation of polymerase, followed by 45 cycles of the 2-step cycling (3 s at 95 °C for denaturation and 30 s at 55 °C for annealing and extension). The assay was conducted in the CFX96 Touch™ Real-Time PCR detection system (Bio-Rad Laboratories) according to the CDC EUA-approved protocol, and the plate read was set at the annealing and extension step.

### 2.7. Clinical Specimens and RNA Isolation

A total of 10, 16, 13, and 10 positive nasopharyngeal specimens for SARS-CoV-2, influenza A and B, and RSV, respectively, and 12 nasopharyngeal specimens that were negative for all the viruses were obtained under the approved protocol HP-00095580. The positive and negative nasopharyngeal swabs were confirmed by clinical RT-PCR diagnostic testing. To eliminate the risk of SARS-CoV-2 transmission, all the specimens were heated at 56 °C for 30 min before for fully inactivating the viruses being shipped to the laboratory. RNA was isolated from the specimens by using the RNeasy kit (Qiagen, Frederick, MD, USA) as described in our previous study [15]. We determined the RNA concentration with a NanoDrop spectrophotometer.

### 2.8. Statistical Analysis

We used Excel’s Solver add-on to determine the limit of detection (LOD) with probit regression and a 95 % confidence interval [16]. The coefficient of variation (CV) was calculated for ten replicates. We determined the limit of quantitation (LOQ) based on multiple experiments’ data to be the lowest nominal concentration where the CV was still below 25%. We also used the standard deviation of the response (Sy) of the curve and the slope of the calibration curve (S) at levels approximating the LOD and LOQ according to the following formulas: LOD = 3.3(Sy/S) and LOQ = 10(Sy/S) [17,18,19,20]. The Sy was determined based on the standard deviation of the y-intercepts of the regression lines [17,18,19,20]. We determined the accuracy of the assays by testing the different dilutions of a positive sample with ten replicates each. The accuracy was expressed as the CV. CV values were accepted when they were less than 20% [21]. We used linear regression to determine the correlation between different methods. We used GraphPad Prism 9 software (Graphpad Inc; San Diego, CA, USA) to perform statistical analysis and plot the graphical presentation. Differences in values were evaluated using a student’s t-test, and *p*-values less than 0.05 were considered statistically significant in all the analyses.

## 3. Results

### 3.1. CRISPR-Cas12a Can Specifically Discriminate the Different Viruses

Several different concentrations of LbaCas12a, gRNA, and ssDNA-FQ reporters were first investigated to determine the optimal condition of the CRISPR-Cas12a assay for the detection of SARS-CoV, influenza A, influenza B, and RSV. The optimized concentrations of the LbaCas12a, gRNA, and ssDNA reporters were 640 nM, 640 nM, and 800 nM, respectively. With the concentrations, the fluorescence intensity of the CRISPR-Cas12a, with the specific gRNAs significantly raised with the increase in reaction time in the samples. Furthermore, the system could produce significant fluorescence values as early as in 5 min.

**Figure 3:**
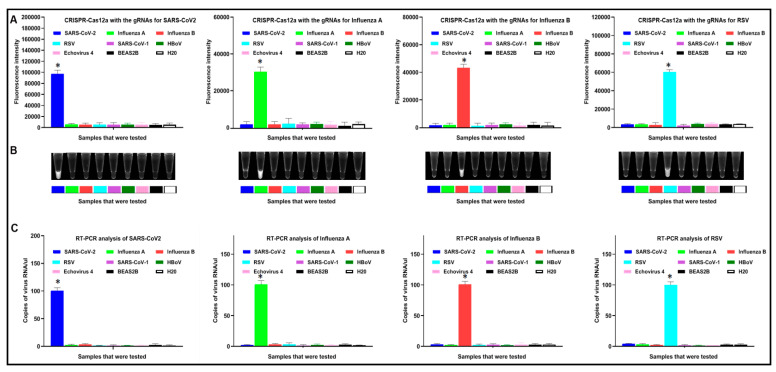
The specificity of CRISPR and RT-PCR for detection of the different viruses. (**A**) The results of CRISPR, represented by fluorescence intensity, were read by a fluorescence plate reader in 10 min. The X-axis shows the RNA sample of each virus. The Y-axis indicates the fluorescence intensity of each sample. The error bars represent the standard deviation from the mean of the fluorescence intensity generated from ten replicates per sample. (**B**) The results of CRISPR are read by a UV transilluminator in 10 min. (**C**) The results of RT-PCR analysis of the same specimens. The X-axis shows the RNA sample of each virus. The Y-axis shows the copy number of the virus RNA measured by RT-PCR in each sample. The error bars represent the standard deviation from the mean of the copy number generated from ten replicates per sample. * *p* < 0.0001.

The RNA of SARS-CoV, influenza A, influenza B, RSV, and other viral nucleic acid extracts (SARS-CoV-1, HBoV, and Echovirus 4) were further subjected to the CRISPR-Cas12a assay for determining the analytic specificity. When the results were read by a fluorescence plate reader, CRISPR-Cas12a with specific gRNA against each virus showed positive results only for the targeted virus without cross-reactivity with other respiratory viruses, including SARS-CoV-1, HBoV, and Echovirus 4 (Figure 3A). Consistently, the visual detection by a UV light illuminator showed that CRISPR-Cas12a could specifically distinguish the different viruses (Figure 3B). For comparison, RT-PCR was conducted in the same sets of samples. The RT-PCR assay displayed the same specificity for differentiating the viruses (Figure 3C).

### 3.2. CRISPR-Cas12a Can Sensitively Detect SARS-CoV-2, Influenza A and B, and RSV

**Figure 4:**
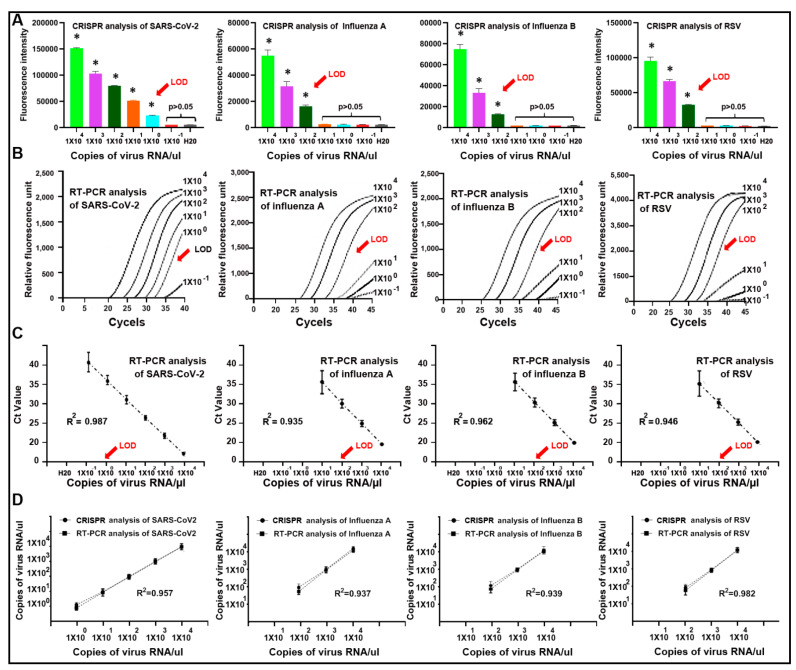
The sensitivity of CRISPR-Cas12a and RT-PCR for detecting SARS-CoV-2, influenza A and B, and RSV in serially diluted RNA standard samples. (**A**) The LOD of CRISPR for detecting the viruses was 1–100 copies/µL (* *p* < 0. 01). The X-axis shows the serially diluted concentrations (from 10^4^ to 0.1 copies/µL) of the RNA standard samples that were tested. The Y-axis indicates the fluorescence intensity of each sample read on the fluorescence plate reader. The error bars represent the standard deviation from the mean of the fluorescence intensity generated from ten replicates in each sample. (**B**) Amplification curves of RT-P CR analysis of the serially diluted RNA samples. Amplification plots were created when the relative fluorescence unit (Y-axis) from each sample was plotted against the cycle number (X-axis). (**C**) The high linearity (R^2^ = 0.902–0.997) of all standard curves of the four types of viruses analyzed by RT-PCR. The X-axis indicates the serially diluted RNA samples that were tested. The Y-axis shows the cycle number of RT-PCR. The error bars represent the standard deviation from the mean of cycle number of RT-PCR generated from ten replicates in each sample. (**D**) Linear regression analysis shows that the CRSIPR and RT-PCR assays had great agreement for detection of the viruses (All R^2^ > 0.90, *p* < 0.01). The X-axis shows the serially diluted RNA samples tested. The Y-axis displays the copies of virus RNA per µL measured by CRSIP or RT-PCR in each of the serially diluted RNA samples. The error bars represent the standard deviation from the mean of copy number generated from ten replicates per concentration.

To determine the sensitivity of the plate-based CRISPR-Cas12a for simultaneously detecting the viruses, whole genomic virus RNA was serially diluted in nuclease-free water and subjected to a CRISPR-Cas12a reaction. The results were read by a fluorescence plate reader. For comparison, the samples were also tested by RT-PCR for the detection of the viruses. CRISPR-Cas12a with specific gRNAs had an LOD of 1 copy/µL for SARS-CoV-2 and 100 copies/µL for influenza A and B and RSV, respectively (Figure 4A). Furthermore, CRISPR-Cas12a with specific gRNAs produced an LOQ of 3 copies/µL for SARS-CoV-2 and 226–348 copies/µL for influenza A and B and RSV, respectively (Supplementary Figure S1). RT-PCR displayed an LOD of 1 copy/µL for SARS-CoV-2 and 100 copies/µL for influenza A and B and RSV, respectively (Figure 4B,C). In addition, linear regression analysis showed that the CRISPR and RT-PCR assays had great agreement in the detection of the viruses (All R^2^ > 0.90, *p* < 0.01) (Figure 4D). The RNase P gene, determined by both CRISPR and RT-PCR, always exhibited a positive signal in all the tested samples. Therefore, CRISPR-Cas12a had comparable sensitivity for detection of the viruses to RT-PCR. Furthermore, the CRISPR-Cas12a results could be automatically generated by immediately and directly reading the fluorescence intensities. However, the RT-PCR results were indirectly calculated by using the cycle threshold (Ct) as a metric, in which the Ct values for the targets were referenced to an internal control gene across samples for normalization. In addition, the plate-based CRISPR-Cas12a took place on a fluorescence plate reader at 42 °C and only required 30 min for the detection of multiple viruses at one time. In contrast, the RT-PCR assay consisted of multiple steps conducted at different temperatures and took more than two hours.

Furthermore, the CV values generated from the different concentrations with ten replicates were <20% (Supplementary Tables S1–S4), suggesting that the CRISPR test could have a high level of accuracy for all the tested dilutions.

### 3.3. Diagnostic Performance of the Plate-Based CRISPR-Cas12a Assay for the Simultaneous Detection and Differentiation of SARS-CoV-2, Influenza, and RSV in Clinical Specimens

The CRISPR-Cas12a assay was conducted for simultaneous detection of the multiple viruses in nasopharyngeal swabs, including 10 positive SARS-CoV-2 (*n* = 10), 16 positive influenza A, 13 positive influenza B, or 12 positive RSV samples, as well as 12 negative nasopharyngeal swabs. The positive and negative nasopharyngeal swabs were confirmed by clinical RT-PCR diagnostic testing, which was used as the gold standard test to determine the efficiency of the CRISPR-Cas12a assay. In each run, the RNase P gene was used as an internal positive control for the experiments. The results in the plate-based CRISPR test were automatically read on a fluorescence plate reader. The mean and standard deviation (SD) of the fluorescence signals of CRISPR-Cas12a in all negative samples were 6,329 (567), 1,735 (148), 2,354 (213), and 1,933 (158) for SARS-CoV-2, influenza A, influenza B, and RSV, respectively. The mean and SD of the fluorescence signals of CRISPR-Cas12a in the positive samples were 93,782 (1,832), 35,648 (1,653), 43,361 (1,980), and 62,379 (1,316) for SARS-CoV-2, influenza A, influenza B, and RSV, respectively. There was a significantly different level of the viruses in the negative specimens compared with the positive specimens (all *p* < 0.001). The RNase P gene exhibited a positive signal across the positive and negative specimens without a statistical difference between the groups. We set a cut-off value for determining positive results by adding 3 SDs on top of the mean of the fluorescence signals obtained from the negative samples. As a result, the cutoff values for positive SARS-CoV-2, influenza A, influenza B, and RSV were 8,030, 2,179, 2,993, and 2,407, respectively. Based on the cutoff values, CRISPR-Cas12a with each specific gRNA could detect SARS-CoV-2, influenza A, influenza B, and RSV in 10 of 10, 15 of 16, 13 of 13, and 9 of 10 corresponding positive specimens, respectively (Table 2). CRISPR-Cas12a did not show positive results in the negative clinical specimens. Therefore, the CRISPR-Cas12a test produced 100.0%, 93.8%, 100.0%, and 90.0% sensitivity for SARS-CoV-2, influenza A, influenza B, and RSV, respectively, with a specificity of 100%.

## 4. Discussion

Nucleic acid, antibody, and protein-based tests have been developed for the detection of SARS-CoV-2, influenza A and B, and RSV, of which RT–PCR for viral nucleic acid detection remains the main approach [22,23]. For instance, the cobas^®^ SARS-CoV-2 & Influenza A/B Nucleic acid test was approved by the Food and Drug Administration (FDA) for emergency use authorization. However, the test requires the expensive cobas^®^ Liat^®^ System. Furthermore, on 12 March 2021, the FDA warned of potential false positive results with the cobas test, since it had an increased likelihood of false positive influenza B results. The Xpert four-in-one assay enables detection of SARS-CoV-2, influenza A, influenza B, and RSV in upper respiratory tract specimens [3,24]. However, it had positive results in the specimens with SARS CoV-1, leading to a false positive result. Reverse transcription loop-mediated isothermal amplification (RT-LAMP) was developed as a simple test for nucleic acid detection of multiple viruses [25]. RT-LAMP requires multiple primers to amplify one target. Since numerous sets of primers for multiple viruses are required in one tube, the formation of primer dimers is often present in the reaction. Therefore, RT-LAMP has lower amplification efficiency and sensitivity compared with the RT-PCR assay [26].

CRISPR-Cas biology has revolutionized the field of molecular diagnostics for various diseases [11]. Previous studies, including our own, have shown that CRISPR could be used for the detection of SARS-CoV-2 [15,27,28,29]. Mayuramart et al. recently used CRISPR to detect SARS-CoV-2, influenza A, and influenza B viruses [30]. However, Mayuramart’s CRISPR test only detects a single target per reaction [30]. It is time-consuming and labor-intensive for the detection of multiple viruses with samples split across multiple reactions for confirmatory detection or controls [30]. Furthermore, the CRISPR test does not detect RSV, which is one of the leading causes of global acute lower respiratory tract infections and has similar symptoms to COVID-19.

By extending our previously developed CRISPR-Cas12a tests for the detection of DNA mutations and viruses, including SARS-CoV-2 [12,13,14,15], herein, we describe an approach for the simultaneous detection and differentiation of SARS-CoV-2, influenza A and B, and RSV. Compared with the previous techniques, this plate-based CRISPR-Cas12a test has some advantages. It has similar sensitivities, compared to the RT-PCR assay, for detection of the viruses. It is highly specific without any cross-reactivity with other respiratory viruses. The reaction takes place in one plate at a constant temperature, thus reducing the possibility of contamination. It also diminishes the number of separate tests and only takes 30 min, thus decreasing the sample turnaround time for the detection of multiple viruses and allowing automated data interpretation. The assay only requires a single 24-well plate and fluorescence plate reader, allows the system to be placed in any lab environment, eliminates user-to-user variability, and reduces reagent usage. The automated workflow can simplify the complexity of combined analysis of different virus types and increase testing throughput. Furthermore, the clinical performance of the assay was validated in nasopharyngeal swabs. Therefore, this CRISPR-Cas12a test would be a clinically useful assay for sensitively detecting and specifically discriminating the different viruses. Furthermore, it has the potential to be developed for simultaneously assessing other important respiratory viruses by using a large microplate (e.g., 486-well plate). This high-throughput assay might represent a robust and novel approach for the prevention of transmission and treatments of the infectious diseases.

The major limitation of this study is that the sample size of the clinical nasopharyngeal swabs was small. A large sample size is needed to prospectively validate the clinical significance of the CRISPR-Cas12a test. Furthermore, only one strain of SARS-CoV-2, influenza A, influenza B, or RSV was tested. We will collect more strains of the viruses and further validate the analytic performance of the CRISPR test. In addition, mutations related to SARS-CoV2 have been reported that are associated with a more transmissible form of the virus [31,32]. We will determine if the Cas12a system with specific gRNAs against the mutations could detect the related variants.

## 5. Conclusions

We developed a simple and high throughput CRISPR-Cas12a test for detecting and distinguishing SARS-CoV-2, influenza A and B, and RSV. Nevertheless, the findings of this study need to be validated in future research with more strains of viruses and large clinical sample studies.

## Figures and Tables

**Table 1:** Sequences for RPA primers.

Primers for RT-RPA	
SARS-CoV2 N-F	TGATTACAAACATTGGCCGCAAATTGCACA
SARS-CoV2 N-R	AGGTCAACCACGTTCCCGAAGGTGTGACTT
Influenza A-F	CTCACTTTTCTAGCACGGT
Influenza A-R	CCACTGGCTACGGCAGGTC
Influenza B-F	GCCATTCGATTTATAGGAAGAGC
Influenza B-R	CACTTGATCAACTAGAGCCT
RSV-F	AGAAATGAAATTTGAAGTGT
RSV-R	GATTCTATCTCAATGTTGAT
RNase P gene-F	TGGAGCCAGAGACCGACACA
RNase P gene-R	ACATGGCTCTGGTCCGAGGT

Abbreviations: RT-RPA = reverse transcription-recombinase polymerase amplification; F = forward primer; and R = reverse primer.

**Table 2:** The diagnostic sensitivity and specificity of CRISPR-Cas12a for detection of the viruses.

Target	The Number of Specimens Tested	The Number of Positive Results	Sensitivity *	Specificity *
SARS-CoV-2	10	10	100.0%	100.0%
Influenza A	16	16	100.0%	100.0%
Influenza B	13	13	100.0%	100.0%
RSV	10	9	90.0%	100.0%

* Clinical RT-PCR diagnostic testing was used as the gold standard.

## Data Availability

The data that support the findings of this study are available from the corresponding author upon a reasonable request.

## Supplementary Materials

The following are available online at https://www.mdpi.com/article/10.3390/diagnostics11050823/s1. Supplementary Figure S1. The LOD and LOQ of CRISPR-Cas12a for detecting SARS-CoV-2, influenza A and B, and RSV in serially diluted RNA standard samples. The standard deviation of the response (Sy) of the curve and the slope of the calibration curve (S) were applied to determine the LOD and LOQ by using the formulas LOD = 3.3(Sy/S) and LOQ = 10(Sy/S). The solid line indicates the LODs, while the dotted line shows the LOQs of the CRSIPR test for detection of each virus. Supplementary Table S1. Accuracy of the CRSPR test for detection of SARS-CoV2. Supplementary Table S2. Accuracy of the CRSPR test for detection of Influenza A. Supplementary Table S3. Accuracy of the CRSPR test for detection of Influenza B. Supplementary Table S4. Accuracy of the CRSPR test for detection of RSV.
