# Supplementary material for: Detection and Differentiation of SARS-CoV-2, Influenza, and Respiratory Syncytial Viruses by CRISPR
Source: Diagnostics (Basel). 2021 May 1;11(5):823. doi: 10.3390/diagnostics11050823 (PMC8147329; doi:10.3390/diagnostics11050823)
Supplement: Supplementary file 1 [file diagnostics-11-00823-s001.zip › diagnostics-1176759-SI.pdf]

Supplementary Figure S1.

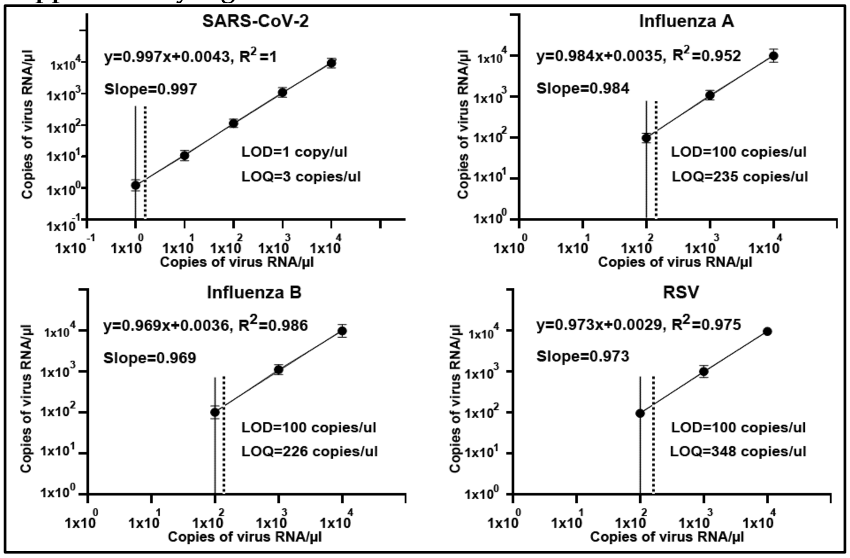

The LOD and LOQ of CRISPR-Cas12a for detecting SARS-CoV-2, influenza A and B, and RSV in serially diluted RNA standard samples. The standard deviation of the response (Sy) of the curve and the slope of the calibration curve (S) are applied to determine the LOD and LOQ by using the formulas: LOD = 3.3(Sy/S) and LOQ = 10(Sy/S). Solid line indicates LODs, while dotted line shows LOQs of the CRSIPR test for detection of each virus, respectively. The Y-axis indicates standard concentrations (copies of virus RNA/µl), X-axis indicate copies of virus RNA/µl measured by the test. Error bars represent the standard deviation from the mean of copies of virus RNA generated from ten replicates in each concentration.

Supplementary S1. Accuracy of CRSPR test for detection of SARS-CoV2

Analyzed dilution samples	Mean	SD	CV (%)*
1 copy/µl	1.052	0.143	13.639
10 copies/µl	9.676	0.569	5.882
100 copies/µl	100.345	5.403	5.385
1,000 copies/µl	1,003.619	15.003	3.695
10,000 copies/µl	9,991.666	106.745	3.287

Supplementary S2. Accuracy of CRSPR test for detection of Influenza A

Analyzed dilution samples	Mean	SD	CV (%)*
100 copies/µl	103.261	5.568	5.392
1,000 copies/µl	1,002.452	11.846	3.182
10,000 copies/µl	10,025.189	151.398	4.510

Supplementary S3. Accuracy of CRSPR test for detection of Influenza B

Analyzed dilution samples	Mean	SD	CV (%)*
100 copies/µl	102.928	8.021	7.793
1,000 copies/µl	1,011.276	16.442	5.640
10,000 copies/µl	10,058.522	204.910	6.037

Supplementary S4. Accuracy of CRSPR test for detection of RSV

Analyzed dilution samples	Mean	SD	CV (%)*
100 copies/µl	103.928	11.590	11.152
1,000 copies/µl	998.119	12.124	4.215
10,000 copies/µl	10,072.855	196.942	5.959

*, CV values are generated from the different concentrations with ten replicates.
